# Medical Updates and Appointment Confirmations: Pet Owners' Perceptions of Current Practices and Preferences

**DOI:** 10.3389/fvets.2019.00080

**Published:** 2019-03-20

**Authors:** Lori Kogan, Regina Schoenfeld, Stacee Santi

**Affiliations:** ^1^Human Animal Interaction Studies, College of Veterinary Medicine and Biomedical Sciences, Colorado State University, Fort Collins, CO, United States; ^2^Veterinary Education and Development, North Carolina State University, Raleigh, NC, United States; ^3^Vet2Pet, Durango, CO, United States

**Keywords:** veterinarian-client communication, text messages, telemedicine, client preferences, medical updates, telehealth, millennials

## Abstract

Pet ownership is increasing, in large part due to the number of millennial pet owners. More pet owners as well as the advent of extensive veterinary care options have resulted in a substantial increase in veterinary care spending. Yet, regardless of client cohort or type of medical procedure performed, communication between clients and veterinarians continues to be a key component in patient care and client satisfaction. Two areas of communication are explored in this study: medical updates to clients when their animals need to stay in the hospital for extended periods of time (at least 4 h) and appointment confirmations. This study, through an anonymous online survey, explored pet owners' stated current modality and frequency of receiving medical updates by their veterinarian and compared these to their stated preferences. Participants' preferences for the modality in which they receive appointment reminders was similarly compared to how they currently receive reminders. There were differences in both frequency (medical updates) and modality (medical updates and appointment confirmations) between what pet owners currently encounter and what they would prefer. In particular, few pet owners receive medical updates or appointment confirmations via text, when a significant portion would prefer this mode of communication. Pet owners also reported wishing to receive medical updates more frequently then they currently experience, with 53.8% of participants reporting they would pay extra for this service. The ramifications of these results are explored with a focus on how to modify these services to best meet the needs of clients.

## Introduction

Pet ownership in the US is increasing - from 80 million pet owning households in 2015 to 84.6 million in 2017 and it has been suggested that millennials (those born between 1980/1981 an d 1995/1996) are a primary factor in driving this growth (1). Millennials are the largest cohort of pet owners and own 35% of US pets (1). They are also more likely to follow veterinary advice compared to older owners (50 vs. 31%) (2). Owners of all generations love their pets with 84% reporting that they love and care for their pet as much as any other family member (3). This can be seen in the lifestyle choices many pet owners make to accommodate their pets. For example, 33% of pet owners' report altering their travel plans to accommodate their pet and 87% worry about their pet while they travel (4). Buying a house is another life choice influenced, for many, by views on what is best for their pet. One study of recent home buyers found that 75% reported they would pass up an otherwise perfect home if it did not meet their pets' needs. This view was endorsed the most by millennials (5). It is not surprising therefore, that millennial pet owners outspend other generations on pet care (6, 7). Pet owners of all ages, however, spent 69.51 billion on their pets in 2017; a 4% growth from 2016. Specifically, veterinary care witnessed a 7% increase between 2016 and 2017 (8). Many of the medical advances in veterinary medicine necessitate extended time at the clinic/hospital (e.g., conditions requiring IV fluid therapy, surgical conditions requiring multiple surgeries, etc.). How veterinary clinics communicate with clients during these times has impact on both patient care and client satisfaction. Communication within veterinary medicine has received increased attention and felt to be a core skill for successful veterinarians (9). The current study was designed to determine how the veterinary field is meeting client needs in the areas of medical updates and appointment confirmation.

## Materials and Methods

### Methodology

An online survey was created in Qualtrics and survey respondents were recruited through Amazon's Mechanical Turk [a crowdsourcing online labor market that coordinates the supply and the demand of cognitive tasks (10)] to assess pet owners' perceptions of medical updates provided by their veterinarian and veterinary appointment reminders. The survey was designed, reviewed, and tested by researchers at Colorado State University (CSU) who provided feedback on content, navigability, and overall questionnaire design. A pilot survey was distributed to ten pet owners who were known to one of the authors (LK). Respondents were asked to complete the online survey and to provide feedback including suggested changes and/or improvements on whether they understood all of the questions and questionnaire format. This resulted in minor changes to the survey and a final questionnaire was drafted. The final questionnaire was deemed exempt by the Institutional Review Board at CSU.

The online survey was posted October 9, 2018–October 29, 2018. The first page of the survey described the survey characteristics (e.g. length, anonymity), ethics approval, optional nature of the survey and voluntary completion/withdrawal process. Participants were screened to ensure they currently owned a dog or cat (or both) and had a pet that required hospitalization for at least 4 h (approximately one-half day). The term hospitalization was used to infer procedures that required veterinary care.

Respondents that met the criteria were asked a series of questions related to medical updates they received during the last time they had a pet stay at the veterinary clinic. They were asked about update frequency and modality, as well as their preference for both frequency and modality for these updates. They were then asked about their willingness to pay for such services. Lastly, they were asked how they received appointment confirmations and their preference for these reminders.

### Statistical Analysis

Data was entered into a statistical software program (SPSS 23 for Windows, Version 22.0. IBM Corp.). Descriptive statistics and Chi Square were used to analyze data. A value of *p* < 0.05 was accepted as significant.

## Results

In accordance with the eligibility criteria, all respondents (*n* = 1,031) owned a dog (460, 44.6%) or cat (246, 23.9%) or both (325, 31.5%) that had to stay in a veterinary hospital for at least 4 h. Most (748; 72.6%) had a pet who required overnight hospitalization, while 283 (27.4%) had a pet who did not stay overnight. Participants were asked to answer the first several questions about their experiences receiving medical updates from their veterinarian based on the last time they had a pet remain hospitalized for at least 4 h.

Owners were asked to report the length of their pets most recent stay at the veterinary clinic (*n* = 1,031). The most common response was 1 night (420, 40.7%), followed by 4–8 h (356, 34.5%), 2 nights (101, 9.8%), more than 2 nights (82, 8.0%) and more than 8 h but not overnight (72, 7.0%).

### Updates

Most owners reported that their veterinary clinic (veterinarian or staff) had given them updates (782, 75.8%) but 249 (24.2%) said they did not receive updates. This was significantly different based on the length of time the pet was in the hospital (chi square 18.98, *p* = 0.001) (Table 1). When asked how often they received updates, most clients reported getting updates once per day (370, 47.6%), followed by 2 times/day (313, 40.2%), 3 times/day (63, 8.1%), more than 3 times/day (23, 3.0%) and other (9, 1.2%). The “other” responses were when the client called (*n* = 7), every 2 h (*n* = 1) and when the owner worked at the hospital (*n* = 1). How often they received updates was significantly different based on the length time the animal spent in the hospital (Chi square 32.0, *p* = 0.010) (Table 2).

**Table 1:** Veterinary updates and time animal spent in hospital (*n* = 1031).

**Time**	**Yes**	**No**
4–8 h	257 (72.2%)	99 (27.8%)
More than 8 h but not overnight	56 (77.8%)	16 (22.2%)
1 night	308 (73.3%)	112 (26.7%)
2 nights	88 (87.1%)	13 (12.9%)
More than 2 nights	73 (89.0%)	9 (11.0%)

**Table 2:** Frequency of updates and time animal spent in hospital (*n* = 1031).

	**1 time/day**	**2 times/day**	**3 times/day**	**More than 3 times/day**	**Other**
4–8 h	**127 (49.8%)**	96 (37.6%)	19 (7.5%)	12 (4.7%)	1 (0.4%)
More than 8 h but not overnight	21 (37.5%)	**25 (44.6%)**	8 (14.3%)	1 (1.8%)	1 (1.8%)
1 night	**140 (45.6%)**	131 (42.7%)	25 (8.1%)	7 (2.3%)	4 (1.3%)
2 nights	34 (39.1%)	**43 (49.4%)**	8 (9.2%)	2 (2.3%)	–
More than 2 nights	**48 (65.8%)**	18 (24.7%)	3 (4.1%)	1 (1.4%)	3 (4.1%)

*Bold values are most common response*.

Participants who reported receiving updates (*n* = 782), were asked to report the modality in which they received these updates (they could select more than one). The primary modality was phone (707, 90.4%), followed by text (104, 13.3%), email (39, 5.0%), video (10, 1.3%), and other (24, 3.1%). The “other” responses consisted of “in person” (17), “photo” (1) “website” (1) “voice mail” (1), “Facebook” (1), “text pictures” (1), and “client returned call” (2). The majority of pet owners reported liking the updates (strongly liked: 403, 51.8%; liked: 293, 37.7%; neutral: 67, 8.6%; disliked: 11, 1.4%; and strongly disliked: 4, 0.5%). When asked to rate the importance of updates, most participants reported that the updates were very important (799, 78.1%), 214 (20.9%) reported they were somewhat important and only 10 (1.0%) reported updates were not important.

Owners were then asked their preference regarding frequency and modality of updates if they had a pet that required a hospital stay of at least 24 h at some point in the future. The most common frequency response was every 4–6 h, followed by every 2–3 h (Table 3). Next, owners were asked to rank their preference regarding how they would like to receive updates. Forty-two percent of clients ranked phone call at home/cell as their first choice, and 38.1% ranked text messages as their first choice (Table 4).

**Table 3:** Preference for frequency of updates (*n* = 1,022).

	**Number**	**Percent**
Hourly	86	8.4
Every 2–3 h	278	27.2
Every 4–6 h	358	35.0
Every 8 h	145	14.2
Every 12 h	96	9.4
Every 24 h	40	3.9
I would not want updates	3	0.3
Other	16	1.6

*Most of the “other” responses wanted updates if something changed or there was something to report*.

**Table 4:** Owner ranking of update modality preference (*n* = 1,023).

	**Ranked 1**	**Ranked 2**	**Ranked 3**	**Ranked 4**	**Ranked 5**
Phone call cell/home	430 (42.0%)	242 (23.7%)	185 (18.1%)	111 (10.9%)	55 (5.4%)
Text	390 (38.1%)	307 (30.0%)	218 (21.3%)	88 (8.6%)	20 (2.0)
Phone at work	91 (8.9%)	202 (19.7%)	159 (15.5%)	261 (25.5%)	310 (30.3%)
Video	64 (6.3%)	74 (7.2%)	141 (13.8%)	235 (23.0%)	509 (49.8%)
Email	48 (4.7%)	198 (19.4%)	320 (31.3%)	328 (32.1%)	129 (12.6%)

The owners who indicated that updates were at least somewhat important to them (*n* = 1,013) were asked how much they might be willing to pay for these services (1,011 responded to this question). The largest percentage said they would not be willing to pay for updates (467, 46.2%) followed by 240 (23.7%) who said they would be willing to pay an additional 3% of the total cost of their pets' procedure, 203 (20.1%) who said they would pay an additional 5%, and 101 (10.0%) who reported they would pay an additional 10%.

Lastly, clients were asked how they receive appointment confirmations (*n* = 10,31), with most indicating they get these via phone messages on their home/cell phone (582, 56.5%), followed by text (260, 25.2%). This can be compared to the responses given when asked about their preference for appointment confirmations, whereby the most common response was text (526, 51.5%), followed by messages on home/cell phone (300, 29.4%) (Table 5).

**Table 5:** Current and preferred update modality.

**Type**	**Current modality for receiving appointment confirmations (*****n*** **=** **1,031)**		**Preferred modality for receiving appointment confirmations (*****n*** **=** **1,021)**	
	**Number**	**Percent (%)**	**Number**	**Percent (%)**
Phone message cell/home	582	56.5	300	29.4
Text	260	25.2	526	51.5
Email	237	23.0	149	14.6
Do not get confirmations/ do not want confirmations	143	13.9	23	2.3
Mail	63	6.1	10	1.0
Phone message at work	31	3.0	13	1.3

## Discussion

The majority of participants reported that their veterinarians provided medical updates when their animal needed to stay in the hospital for an extended period of time. The frequency of these updates was most commonly 1–2 times a day with a trend toward less frequent updates for animals hospitalized more than two nights. The number of updates owners received, regardless of how long their animal was in the hospital, appears to be lower than they would have preferred. Over one-third of owners indicated they would like updates every 2–3 h and an additional 35% reported they would like updates every 4–6 h. Only 3.9% reported wanting updates every 24 h. It is noteworthy that 53.8% of respondents said they would be willing to pay a premium for this service. Offering frequent medical updates can be time consuming and challenging for many veterinary practices. Yet, the fact that many clients are willing to pay for this service opens up sustainable and practical avenues in which to offer this service, even within a busy practice.

This discrepancy between owner preference and current practice is seen in the modality used for medical updates. Over 90% of owners reported receiving updates by phone while only 13.3% reported receiving texts. While the top mode for receiving updates as ranked by owners was phone (ranked number one by 42%), this was followed closely by texting, which was ranked number one by 38.1% of owners. It would appear that a significant number of owners would prefer text messages, yet are receiving phone calls. The reasons behind the limited use of texting by veterinarians to provide medical updates is worthy of further exploration. This is especially true since text messages (one-way communication) often take less time than interactive telephone calls.

The minimal use of texting is also evident in how veterinary clients report they receive appointment confirmations. Over half (56.5%) of participants reported receiving appointment confirmations by phone yet only 29.4% said this was their preference. Similarly while only 25.2% of owners reported receiving confirmations by text, 51.5% reported this was their preference.

## Conclusion

This study illuminates several important areas of discrepancy between veterinarians' current client communication practices in terms of modality and frequency of medical updates and appointment confirmations—and clients' preferences. It would appear that while veterinarians are still primarily using verbal communication via the telephone to communicate with their clients, many pet owners would prefer text messages.

Given the ubiquitous nature of cellphones and exponential growth of texting, these results are not surprising. Ninety-five percent of all Americans own a cellphone and 77% own a smartphone. Gender differences are slight (80% of men own a smartphone compared to 75% of women) but age plays a larger role. Whereby 94% of US adults 18–29 own a smartphone, this number decreases to 46% of adults 65 and older (17). This translates into 98% of millennials ages 18–24 owning a smartphone, followed closely by millennials ages 25–34 at 97% (11). One of the primary uses of smartphones is texting. In 2010, 72% of US adults reported texting, with an average of 10 texts per day (17). In fact texting is the most common form of communication for US adults under 50 years of age (18).

Research has demonstrated that text messages can be an effective reminder for improving appointment and medical compliance among adults and adolescents (12). Text messages have also been shown to increase medication adherence (13). Texting can be seen as part of the bigger umbrella of telehealth—the use of technology to deliver health information, education and care and specifically telemedicine in which medical information is exchanged electronically (AVMA, https://www.avma.org/PracticeManagement/telehealth/Pages/telehealth-basics.aspx). As the AVMA report (14) stated, telemedicine is a tool that can enhance animal care by facilitating communication among other things.

Yet, veterinarians have been slow to adopt many aspects of telehealth (e.g., telemonitoring, telemedicine, etc.) for a variety of reasons including legal concerns and lack of time or interest to implement change (15). At the same time, in the results from the Connected Patient Report (16) a majority of US medical patients want communication with their physician to include digital options like texting and two-way videos, veterinary clients desire alternative ways to communicate with their veterinarian.

This is perhaps especially true for millennial pet owners. These owners, according to the American Pet Products Association, have the desire to offer their animals optimal care and prefer to partner with their veterinarians. They follow veterinary advice, but they also are likely to obtain information from additional sources beyond veterinary clinics (2). Regardless of where they obtain their information, they value convenient and easily accessible communication options. In fact, millennials rated chat or texting as the veterinary service they value most (2). Given the growing use of texting, this is not surprising. As with other elements of telehealth, it is imperative that veterinarians be willing to critically evaluate their current practices and update them as needed to best meet the needs of their clients. This might be especially true for millennial pet owners, who are poised to significantly impact veterinary medicine.

Limitations of the current study include a sample that may not be representative of US pet owners. It is important to note that because this study was conducted online, the results may not generalize to all pet owners. Further research with pet owners obtained via other avenues is warranted. Additionally, the age of pet owners and their geographical location (e.g., urban, rural) are factors worth further exploration as they may impact pet owners' views on both frequently and modality of medical updates. In conclusion, this study highlights two services that veterinary practices can improve upon—medical updates and appointment confirmations—to better meet the needs of their clients.

## Data Availability

The datasets generated for this study are available on request to the corresponding author.

## Author Contributions

LK and RS conceived the study, conducted the research, and wrote the manuscript. SS reviewed research design and helped prepare the manuscript.

### Conflict of Interest Statement

The authors declare that the research was conducted in the absence of any commercial or financial relationships that could be construed as a potential conflict of interest.
